# Supplementary material for: Multi-Resin Masked Stereolithography (MSLA) 3D Printing for Rapid and Inexpensive Prototyping of Microfluidic Chips with Integrated Functional Components
Source: Biosensors (Basel). 2022 Aug 17;12(8):652. doi: 10.3390/bios12080652 (PMC9405740; doi:10.3390/bios12080652)
Supplement: Supplementary file 1 [file biosensors-12-00652-s001.zip › biosensors-1858554-SI.pdf]

Supplementary Information

Multi-resin masked stereolithography (MSLA) 3D printing for rapid and inexpensive prototyping of microfluidic chips with integrated functional components

Isteaque Ahmed[a], Katherine Sullivan[a] and Aashish Priye[a]

[a]Department of Chemical and Environmental Engineering, University of Cincinnati, Cincinnati, Ohio, United States

Contents

A. SLA printer specification comparison ... 2
B. Optimization of resin exposure time and printer parameters ... 2
C. Testing the reliability of printing embedded channels ... 2
D. Changing exposure times for multi-material print ... 3
E. Effect of print orientation on print quality ... 4
F. Tensile stress testing device: ... 4
F.1. Mechanical: ... 4
F.2. Electrical: ... 4
F.3. Software: ... 5
G. Nucleic acid amplification test in 3D printed resin ... 6
H. References ... **Error! Bookmark not defined.**

A. SLA printer specification comparison

Table S1: Specifications of masked-stereolithography printers used in this study

	Prusa Sl1	Elegoo Mars 3 4K	Elegoo Mars 2 mono
Screen size	5.5 (inch)	6.6”	6.08 (inch)
Curing wavelength	405nm	405nm	405nm
LCD type	RGB	Monochrome	Monochrome
XY resolution	2560x1440	4098x2560	2560*1620
Z resolution	10 µm	10 µm	10 µm
Pixels per square inch (PPI)	479	726	509
Pixel size	47 µm	35 µm	47 µm
Build volume (maximum)	120 × 68 × 150	143 × 90 × 165	129 × 80 × 160
File type	.sl1	.ctb	.ctb (v3)
Native slicer	Prusa slicer	Chitubox	Chitubox
Bed tilt	yes	no	no
Ability to change exposure during print	no	yes	yes
Pause mid-print	no	Yes	yes
Price	$2000	$350	$200

B. Optimization of resin exposure time and printer parameters

Table S2: Resin optimum exposure times for commercial and custom resins used in this study

Resins	[EGPEA(): PEGDA()]	IRG 819 (% wt)	NPS (% wt)	Exposure times* (s)	Comment
Siraya tech	-	-	-	1.25	Though it has very good transparency, the channels clog up at 400x400 µm cross section.
Shine sing	-	-	-	1.15	The printed parts were relatively translucent and hazy.
Anycubic	-	-	-	1.3	Appearance of yellow hue on the printed parts.
resin 1	[1:0]	1	1	2.25	Extremely flexible printed parts
resin 2	[1:0.1]	1	1	2.25	
resin 3	[1.0.3]	1	1	2.25	
resin 4	[1:0.5]	1	1	2.25	
resin 5	[1.0.7]	1	1	2.25	
resin 6	[1:1]	1	1	2.25	Rigid printed parts.
resin 7	[0.7:1]	1	1	2.25	
resin 8	[0.5:1]	1	1	2.25	

*Minimum exposure time, below which the part doesn’t print.

C. Testing the reliability of printing embedded channels

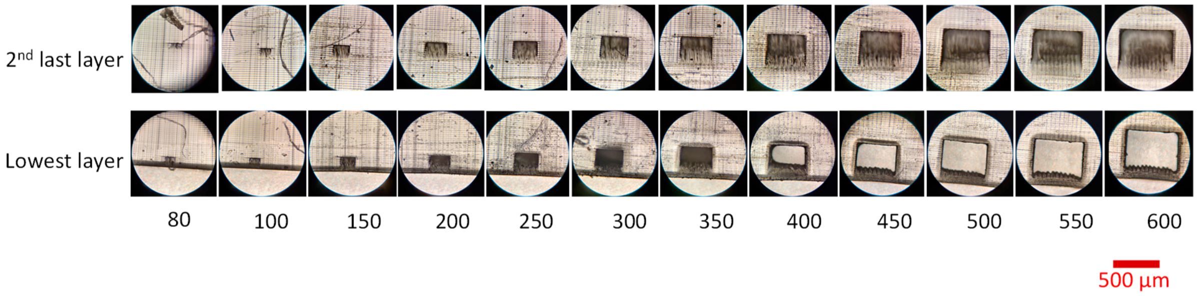

Figure S1: Embedded channel cross-section using commercial resins (Anycubic clear tough)

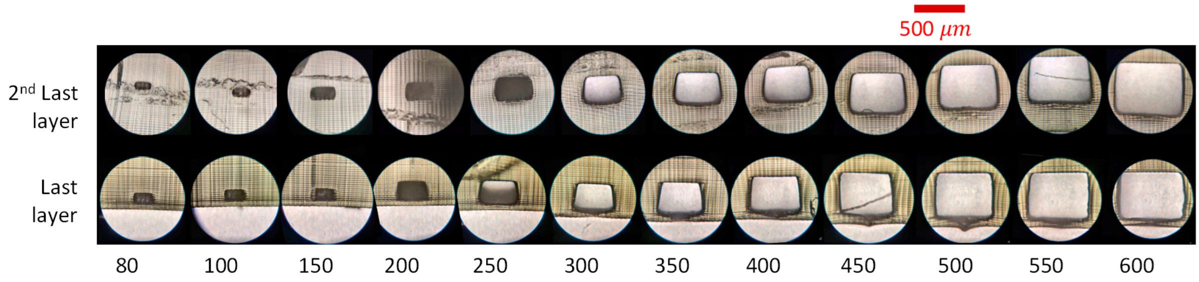

Figure S2: Embedded channel cross-section using the custom resin (EGPEA:PEGDA 1:1)

D. Changing exposure times for multi-material print

Changing resins by pausing a print job requires changing the exposure to match the initial exposure time for that specific resin. Since the printers aren't designed to pause in this manner, this process needs to be done manually. Matching the exposure (both initial and normal exposure) depends on the type of the resin and the layer it is supposed to be changed on.

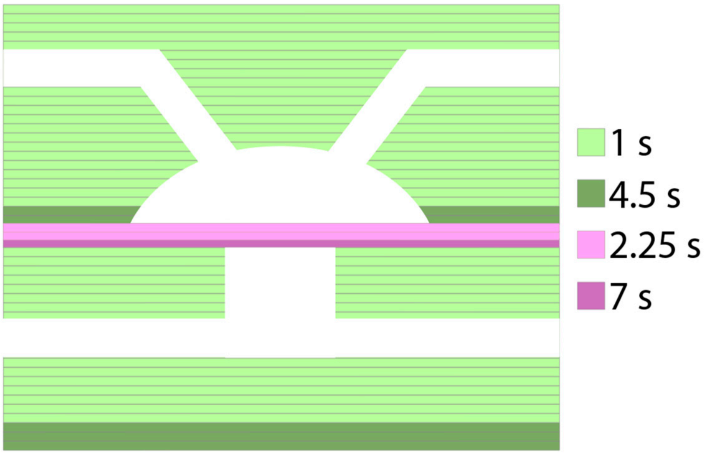

Figure S3: Changing the exposure while printing multi-material device. The print starts from the bottom layer and ends at the top layer. Each material change should go through same exposure cycle as shown.

E. Effect of print orientation on print quality

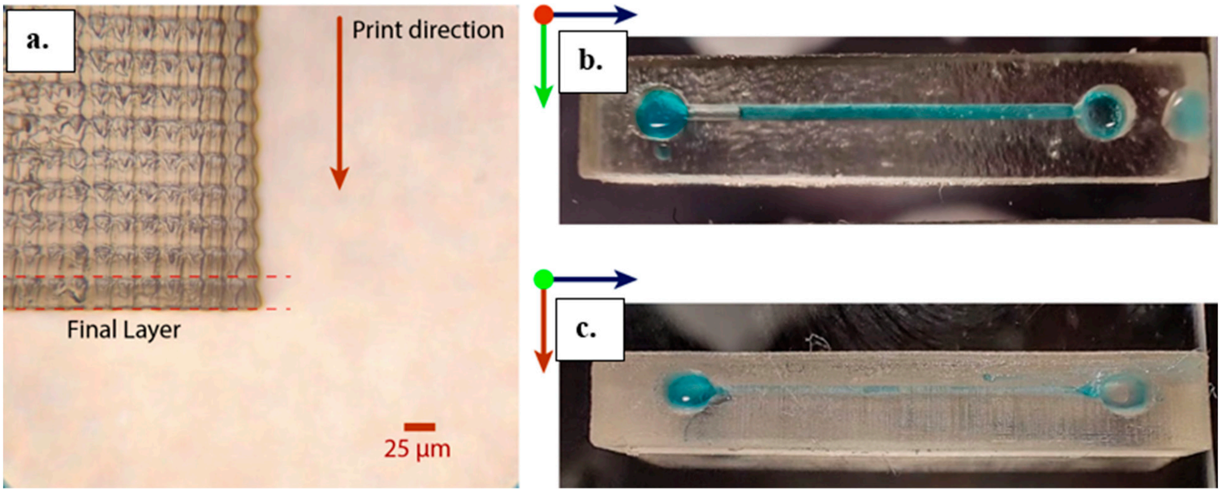

Figure S4: a) microscope image of side view of a printed part edge. A straight embedded channel printed with print direction b) parallel to the x-y plane, c) normal to the x-y plane

F. Tensile stress testing device:

The stress testing apparatus is based on the concept of compression and tension. We designed a device that applies tensile force to the object (cured resin) until it breaks. A stepper motor connected to a lead screw followed by a load cell pulls the clamp holding the cured parts. The load cell provides the reading for the applied force from which Young's modulus and yield stress can be calculated.

F.1. Mechanical:

The whole device is based on an aluminum extrusion platform (2060 EU standard aluminum extrusion base) allowing us to attach other custom components with M4 bolts. There's a stationary clamp and a moving clamp. A load cell is attached to the moving clamp followed by a lead screw connected with a Nema-17 stepper motor. The lead screw sits coaxially with the motor and the center of the clamp. We used an S-shaped load cell (S Type Beam Load Cell; 500 kg Pull Pressure Sensor; Cat #: Walfrontgze5whfktb) which is sufficient to probe the tensile properties of the cured polymer resins.

F.2. Electrical:

We used an Arduino Nano microcontroller as the central processing unit for the whole system. The stepper motor and the load cell were connected to the Arduino microcontroller in conjunction with a stepper motor driver (adafruit; A8988) and a load cell amplifier (adafruit; HX711) respectively. The Arduino board controls the stepper motor movement as well as generating the load cell output simultaneously. A 12V DC (10-amp maximum current) power supply was used as a power source.

F.3. Software:

A custom Arduino code was used to upload the commands from the computer to the Arduino microcontroller. To store and analyze the load cell data, Python's native Arduino package was used. A custom python script was used to generate the stress-strain plots.

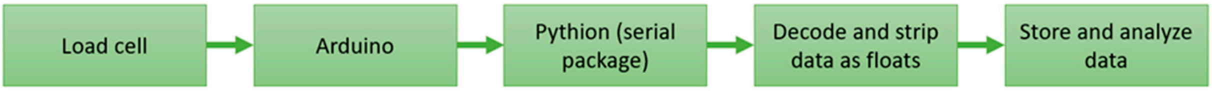

Figure S5: Flow diagram of the primary steps for setting up the load cell and collecting the data.

Table S3: Specifications of masked-stereolithography printers used in this study

Part type	*Part*
Mechanical	Aluminum Extrusions base
	Carriage and rails (MGN12H)
	Shaft collar (12mm)
	Coupler (12mm)
	S-type load cell (for 500g/1kg)
	Bolts and nuts for the assembly
Electrical	Breadboards
	Arduino nano
	Load Cell Amplifier HX711 board
	Nema 17 Stepper motor
	Stepper motor driver (A9488)
	Breadboard jumper wires
	12V power supply
Software	Arduino app
	Python (with Arduino package)

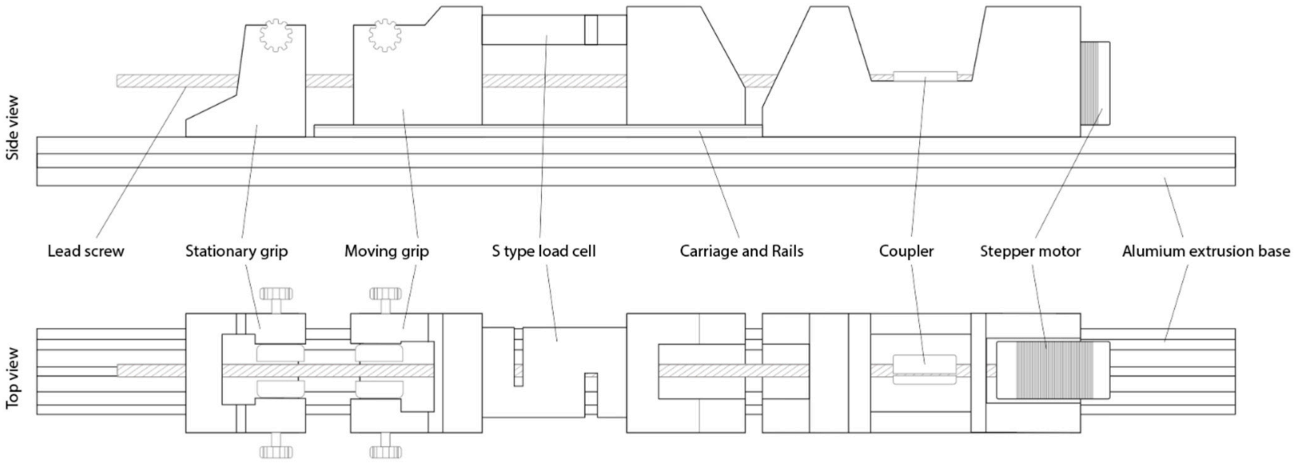

Figure S6: Side view and top view of the apparatus

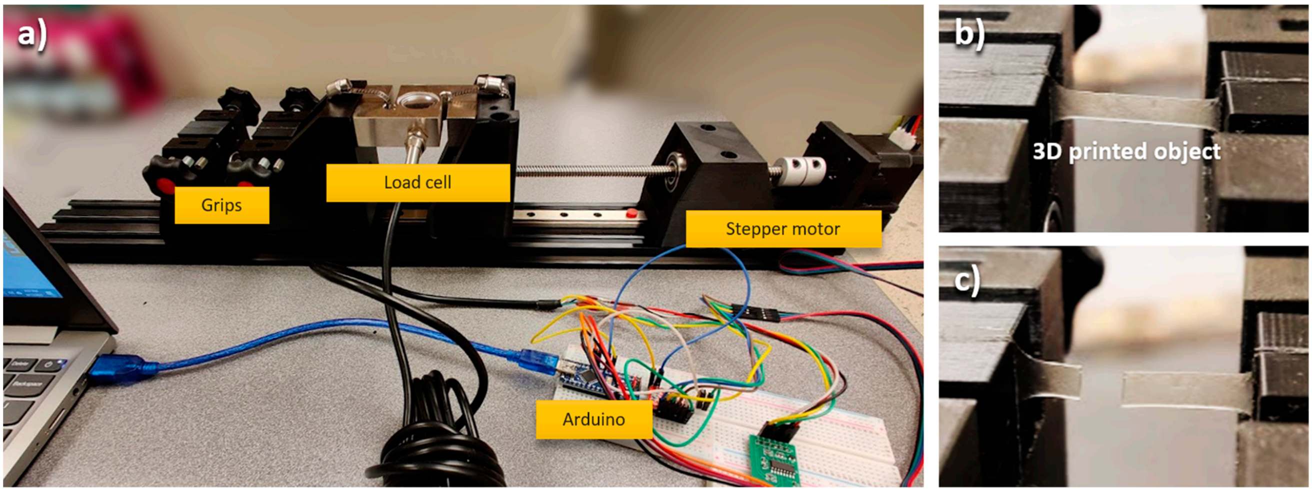

Figure S7: a) apparatus for mechanical stress test of the printed models. b) 3d printed "dog-bone" shaped models using custom resin. c) snapping point

G. Nucleic acid amplification test in 3D printed resin

For testing the biocompatibility of the consumer-resins, we conducted colorimetric LAMP validation of a sample λ DNA. The objective was to SLA print a reaction chamber that can withstand the thermal cycle and produce the colorimetric result otherwise done in a transparent plastic tube. We used 2X master mix protocol (WarmStart Colorimetric LAMP master mix; New England Biolabs) for the lamp reaction and compared the result with a control sample.

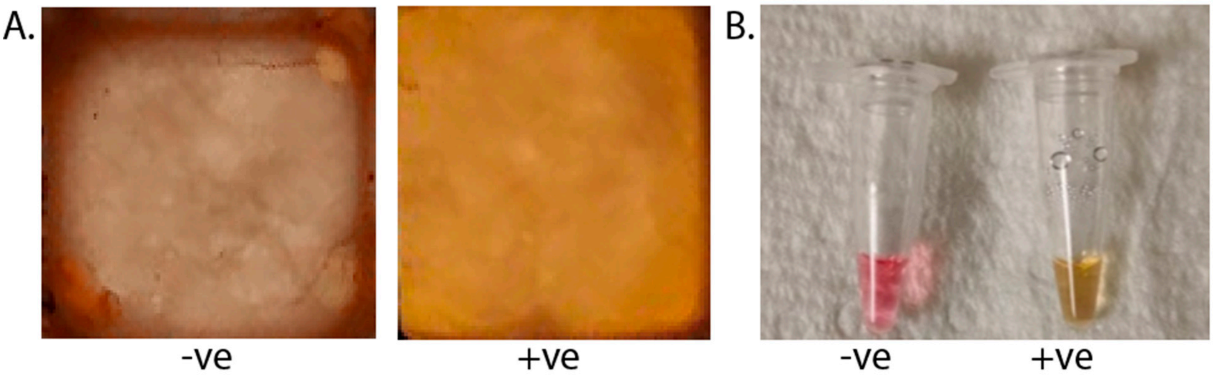

Figure S8: Colorimetric LAMP validation of Lamda DNA. A. Photograph of the amplified DNA sample using 3D printed reaction chamber. B. Traditional tube based colorimetric LAMP validation for Lamda DNA,
